# Use and Spending on Medical Equipment Among US Cancer Survivors

**DOI:** 10.1001/jamanetworkopen.2024.55941

**Published:** 2025-01-22

**Authors:** Faraz I. Jafri, Vishal R. Patel, Syed M. Qasim Hussaini, Naveen Premnath, Helen M. Parsons, Anne H. Blaes, Arjun Gupta

**Affiliations:** 1 University of Texas Health Science Center at Houston; 2 Harvard Medical School, Boston, Massachusetts; 3 Brigham and Women’s Hospital, Boston, Massachusetts; 4 University of Alabama at Birmingham; 5 Division of Hematology, Oncology, and Transplantation, University of Minnesota Twin Cities, Minneapolis; 6 Division of Health Policy and Management, School of Public Health, University of Minnesota Twin Cities, Minneapolis

## Abstract

This survey study assesses patterns of use and costs of medical equipment among US cancer survivors.

## Introduction

Cancer survivors are increasingly older and experience high rates of functional impairment, comorbidity, and complications,^1^ often necessitating medical equipment. Administrative burdens and issues with payer coverage may limit access and increase cost-sharing responsibilities for patients.^2^ We sought to characterize patterns of use and costs of medical equipment among US cancer survivors.

## Methods

This survey study used public, nonidentifiable data that did not constitute human participants research (45 CFR §46.102) and was not submitted for institutional review board review; the reporting followed the AAPOR reporting guideline. We identified adults with self-reported cancer in 2 nationally representative surveys (the National Health Interview Survey [NHIS], 1999-2018 and the Medical Expenditure Panel Survey [MEPS], 2016-2020).

Using NHIS, we evaluated yearly trends in the proportion of cancer survivors who used medical equipment, defined as self-reported use of items including canes, wheelchairs, specialized beds, or specialized telephones. The prevalence of equipment use was adjusted for age, sex, race, and ethnicity using logistic regression. Using MEPS, we evaluated mean inflation-adjusted total and out-of-pocket (OOP) expenditures, and proportion of OOP expenditures (ie, OOP responsibility) related to medical equipment (eMethods in Supplement 1). We used 2-part models to adjust mean expenditure estimates for age, sex, race, and ethnicity, and account for the skewed distribution of expenditure data. For comparison, we extracted expenditures for other medical services (eg, medications). Data analysis was conducted from February to October 2024 using Stata BE version 18.0 (StataCorp). The threshold for statistical significance was a 2-sided *P* < .05.

## Results

**Figure 1.**
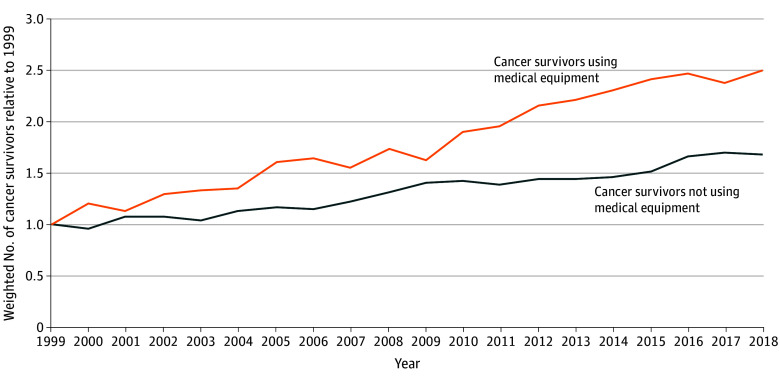
Trends in the Number of Cancer Survivors Reporting Use of Medical Equipment in the US, National Health Interview Survey, 1999 to 2018 Values represent the weighted number of cancer survivors each year relative to those in 1999. Estimates were weighted to account for the National Health Interview Survey’s complex survey design.

From NHIS, we identified 51 258 cancer survivors, representing 17.9 million weighted individuals each year (10.2 million [57.1%] female; 9.1 million [50.7%] older adults aged ≥65 years) from 1999 to 2018. The weighted number of survivors using equipment increased from 1.6 million in 1999 to 4.0 million in 2018, a 2.5-fold increase. The number of survivors who did not use equipment increased by 1.7-fold (Figure 1). The adjusted prevalence of equipment use increased from 6.6% in 1999 to 8.6% in 2018, a 2–percentage point increase (*P* for trend < .001).

**Figure 2.**
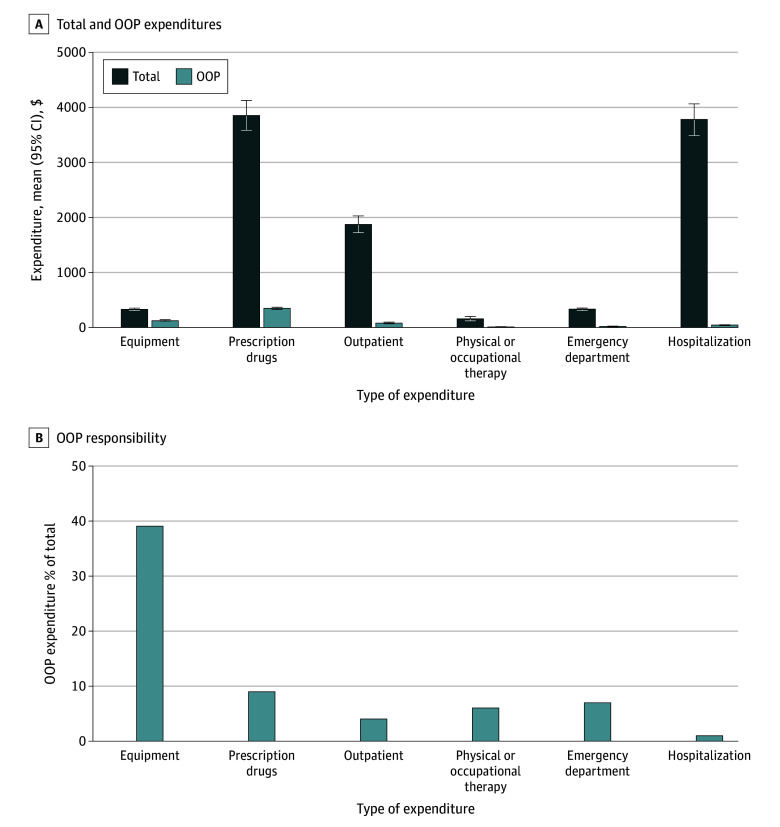
Total and Out-of-Pocket (OOP) Expenditures Among US Cancer Survivors by Type of Health Service, Medical Expenditure Panel Survey, 2016-2020 All estimates were derived using 2-part models that adjust for age, sex, race, and ethnicity. The first part of each model was a probit model of the probability of any annual medical expenditures and the second part of the models were generalized linear models with a γ distribution to account for the skewed distribution of expenditure data. Marginal standardization from the fitted model were used to derive adjusted values. Error bars indicate 95% CIs.

From MEPS, we identified 12 436 cancer survivors, representing 27.9 million weighted individuals each year (15.7 million [56.6%] female; 15.6 million [55.9%] aged ≥65 years) from 2016 to 2020. The mean (SD) annual spending on equipment per survivor was $330 ($11), of which $130 ($6) was OOP, corresponding to a mean annual OOP responsibility of 39% (Figure 2). Mean annual OOP responsibility was lower for prescription drugs (9% [$352 OOP of $3853 total]), outpatient care (4% [$83 OOP of $1873 total]), physical and occupational therapy (6% [$10 OOP of $157 total]), emergency care (7% [$23 OOP of $336 total]), and hospitalization (1% [$46 OOP of $3776 total]).

## Discussion

This survey study found that the number of cancer survivors relying on medical equipment has more than doubled in the past 2 decades, and this category now carries the highest proportion of OOP expenditures across medical services. Despite increases in use, the observed 7.9% prevalence of equipment use in 2018 was much lower than previously reported rates of mobility disability (27.9%) among cancer survivors,^3^ indicating issues with access. In another survey,^4^ 15% of community-dwelling adults younger than 65 years with disability (only 9% were uninsured) delayed or deferred medical equipment use. Common reasons included payer coverage or authorization and affordability issues. Almost one-half of hospital case managers report difficulties obtaining equipment for Medicare beneficiaries.^2^

High equipment costs and OOP responsibility relate to their often urgent clinical need (high willingness to pay), fee schedule (less responsive to competitive market dynamics), and waste and fraud.^5^ Our findings follow 2022 National Health Expenditure Account data, in which the OOP responsibility was 41.5% for medical equipment and 14.0% for prescription drugs.^6^

Study limitations include recall bias, varying definitions of equipment (eg, ambulance in MEPS), question framing (eg, “Are you required to use equipment?” which may underestimate true needs), limited clinical information, and noncapture of nonreceipt of necessary or prescribed equipment. Despite extensive work on cancer-related financial toxicity, the high and often unmet costs of medical equipment remain underrecognized. Policies that improve payer coverage, streamline authorization processes, and reduce cost-sharing burdens for medical equipment are urgently needed.

## Notes

Supplement 1.
eMethods.

Supplement 2.
Data Sharing Statement
