# Supplementary material for: Use and Spending on Medical Equipment Among US Cancer Survivors
Source: JAMA Netw Open. 2025 Jan 22;8(1):e2455941. doi: 10.1001/jamanetworkopen.2024.55941 (PMC11755190; doi:10.1001/jamanetworkopen.2024.55941)
Supplement: Supplement 1. — eMethods. [file jamanetwopen-e2455941-s001.pdf]

Supplemental Online Content

Jafri F, Patel VR, Qasim Hussaini SM et al. Use and spending on medical equipment among US cancer survivors. *JAMA Netw. Open*. 2025;8(1):e2455941. doi:10.1001/jamanetworkopen.2024.55941

eMethods.

This supplemental material has been provided by the authors to give readers additional information about their work.

© 2025 Jafri F et al. *JAMA Network Open*.

eMethods.

“Equipment” definition per NHIS
EQUIPMENT indicates whether sample adults and sample children had any health problem [or, for sample children, "any impairment") that required them to use special equipment. **"Special equipment"** was defined by example in the question wording, **with "such as a cane, a wheelchair, a special bed, or a special telephone"** integrated into the question for sample adults, and "such as a brace, a wheelchair, or a hearing aid (excluding ordinary eyeglasses or corrective shoes) integrated into the question for sample children.

‘’Do you now have any health problem that requires you to use special equipment, such as a cane, a wheelchair, a special bed, or a special telephone?”

NHIS also included a detailed description of special equipment from the field representative’s manual which was not shared with respondents:
"[S]pecial equipment' is **any device, tool, utensil, instrument, implement, etc., used as an aid in performing an activity because of a physical, mental or emotional problem**." The Manual for 1997-2000 continued, "**This includes the use of adult 'diapers' for incontinence**. However, ordinary eyeglasses and hearing aids should not be considered 'special equipment.' For example: a spoon is not normally considered as 'special equipment'; however, a uniquely designed or functioning one used for eating by a person because of physical, mental, or emotional problems is considered 'special equipment.'**"**

“Other medical equipment and services” definition per MEPS
The [equipment and services] category includes **expenditures for ambulance services, orthopedic items, hearing devices, prostheses, bathroom aids, medical equipment, disposable supplies, alterations/modifications, and other miscellaneous items or services that were obtained, purchased, or rented during the year**. Diabetic supplies and insulin are not considered to be medical equipment, and are therefore not included in this variable.

“Hospitalization expenditure” definition per MEPS
The [hospitalization] includes payments for hospital facility expenses **(including direct hospital care, including room and board, diagnostic and laboratory work, x-rays, and similar charges, as well as any physician services included in the hospital charge)** and "separately billing doctor" or SBD expenses **(including services provided to patients in hospital settings by providers like radiologists, anesthesiologists, and pathologists, whose charges are often not included in hospital bills)**.

“Prescription drug expenditure” definition per MEPS
The [prescription drug] category includes the **sum of direct payments made during the year for prescribed medications, including out-of-pocket payments and payments by private insurance, Medicaid, Medicare, and other sources.** Payments for over-the-counter drugs and indirect payments not related to specific medical events, such as Medicaid Disproportionate Share and Medicare Direct Medical Education subsidies, are not included in this amount.

“Emergency room expenditure” definition per MEPS

© 2025 Jafri F et al. *JAMA Network Open*.

The [emergency room] category includes direct payments for care provided during the year for all visits to emergency room medical providers, including out-of-pocket payments and payments by private insurance, Medicaid, Medicare, and other sources.

“Physical and occupational therapy expenditure” definition per MEPS
The [physical and occupational therapy] category includes the sum of direct payments for outpatient care provided during the year, including out-of-pocket payments and payments by private insurance, Medicaid, Medicare, and other sources.

“Outpatient care expenditure” definition per MEPS
The [outpatient care] category includes the sum of direct payments for care provided during the year for all visits to ambulatory occupational and physical therapists, including out-of-pocket payments and payments by private insurance, Medicaid, Medicare, and other sources.

© 2025 Jafri F et al. *JAMA Network Open*.
