# Supplementary material for: Use and Spending on Medical Equipment Among US Cancer Survivors
Source: JAMA Netw Open. 2025 Jan 22;8(1):e2455941. doi: 10.1001/jamanetworkopen.2024.55941 (PMC11755190; doi:10.1001/jamanetworkopen.2024.55941)
Supplement: Supplement 2. — Data Sharing Statement [file jamanetwopen-e2455941-s002.pdf]

Data Sharing Statement

Jafri. Use and Spending on Medical Equipment Among US Cancer Survivors. *JAMA Netw Open*. Published January 22, 2025. doi:10.1001/jamanetworkopen.2024.55941

Data
Data available: No

Additional Information
Explanation for why data not available: The data used in the study are publicly available from the National Health Interview Survey and the Medical Expenditure Panel Survey. Summary data and baseline characteristics of the study sample are available from the corresponding author on request.
